# Can the reform of integrating health insurance reduce inequity in catastrophic health expenditure? Evidence from China

**DOI:** 10.1186/s12939-020-1145-5

**Published:** 2020-04-03

**Authors:** Jiahui Wang, Hong Zhu, Huan Liu, Ke Wu, Xin Zhang, Miaomiao Zhao, Hang Yin, Xinye Qi, Yanhua Hao, Ye Li, Libo Liang, Mingli Jiao, Jiao Xu, Baohua Liu, Qunhong Wu, Linghan Shan

**Affiliations:** 1 Department of Social Medicine, Health Management College, Harbin Medical University, 157 Baojian Road, Nangang District, Harbin, 150086 Heilongjiang Province China; 2 Tong Zhou District’s Volunteer Services Guidance Center of Beijing Municipality, Beijing, China; 3 Department of Health Management, School of Public Health, Nantong University, Nantong, Jiangsu Province China

**Keywords:** Health insurance, Integration, Catastrophic health expenditures, Inequity

## Abstract

**Background:**

China’s fragmentation of social health insurance schemes has become a key obstacle that hampers equal access to health care and financial protection. This study aims to explores if the policy intervention Urban and Rural Residents Basic Medical Insurance (URRBMI) scheme, which integrates Urban Resident Basic Medical Insurance (URBMI) and New Rural Cooperative Medical Scheme (NCMS), can curb the persistent inequity of catastrophic health expenditure (CHE) and further analyses the determinants causing inequity.

**Methods:**

Data were derived from the Fifth National Health Service Survey (NHSS). A total of 11,104 households covered by URRBMI and 20,590 households covered by URBMI or NCMS were selected to analyze CHE and the impoverishment rate from medical expenses. Moreover, the decomposition method based on a probit model was employed to analyse factors contributing CHE inequity.

**Results:**

The overall incidence of CHE under integrated insurance scheme was 15.53%, about 1.10% higher than the non-integrated scheme; however, the intensity of CHE and impoverishment among the poorest was improved. Although CHE was still concentrated among the poor under URRBMI (CI = -0.53), it showed 28.38% lower in the degree of inequity. For URRBMI households, due to the promotion of integration reform to the utilization of rural residents’ better health services, the factor of residence (24.41%) turns out to be a major factor in increasing inequity, the factor of households with hospitalized members (− 84.53%) played a positive role in reducing inequity and factors related to social economic status also contributed significantly in increasing inequity.

**Conclusion:**

The progress made in the integrated URRBMI on CHE equity deserves recognition, even though it did not reduce the overall CHE or the impoverishment rate effectively. Therefore, for enhanced equity, more targeted solutions should be considered, such as promoting more precise insurance intervention for the most vulnerable population and including costly diseases suitable for outpatient treatment into benefit packages. Additionally, comprehensive strategies such as favourable targeted benefit packages or job creation are required for the disadvantaged.

## Introduction

The heavy economic burden of disease has become a major issue for the global health system. A survey of 89 countries by the World Health Organization (WHO) showed that the ratio of people suffering from serious financial difficulties due to illness is increasing by 11% per year, with 5% of the population becoming impoverished due to medical care payments [1]. The catastrophic health expenditure (CHE) index is a critical measure that not only quantifies the economic burden faced by households when out-of-pocket (OOP) health expenses exceed their capacity to pay [2] but also reflects the degree of medical insurance schemes’ financial protection. The rising incidence of CHE has attracted global attention.

Globally, the situation looks grim; statistics from WHO showed that there are 150 million people suffering financial catastrophe, and nearly 100 million individuals are impoverished [1], especially those in low- and middle-income countries [3, 4]. The high incidence of CHE resulted from high poverty rates or a lack of risk-sharing mechanisms [3, 5].

China, as the largest developing country in the world, has a substantial number of households affected by CHE. Li’s study showed that, as of 2008, CHE occurred approximately in 13% of the population [6]. By the end of 2010, there were three types of basic health insurance that had been established in China. First, the Urban Employee Basic Medical Insurance (UEBMI), launched in 1998, was provided mandatorily for employees in urban areas (including retired and rural-to-urban migrant workers), whose premium is to be borne by both the employer and the employee with a combined individual account and socially pooled fund. Individual accounts are mainly used for general outpatient services or to purchase drugs, while the socially pooled accounts are mainly used to pay for inpatient services and some outpatient services for special diseases. Second, in 2003, the New Rural Cooperative Medical Scheme (NCMS) was launched as a voluntary system of mutual assistance through risk pooling to mitigate unaffordable health services and financial burden in rural areas. The funding is from the contribution of the individual and the government; the latter usually accounts for a larger share (70–80%) [7]. The NCMS emphasises the protection against catastrophic illnesses and aims at increasing equity in health care for rural residents. Third, the Urban Resident Basic Medical Insurance (URBMI), launched 4 years after NCMS, was designed for urban residents not covered by UEBMI or NCMS, including primary and secondary school students, young children, and other unemployed urban residents. It is on a voluntary basis at the household level, and sponsored by the government and the individual. URBMI seeks to minimise impoverishment owing to large medical expenditures by focusing on inpatient or outpatient services for chronic or fatal diseases.

Together, these three schemes have covered over 1.35 billion people, participation rate stabilized at more than 95% by 2018 [8]. After the establishment of these health insurance schemes, the major financial barrier hindering smooth access to health services has been lifted, and the ratio of OOP payments to total health expenditures has sharply decreased [9]. However, the benefit packages under URBMI and NCMS could not meet the increasing demands for health services; there are still many residents who lacked access to health care because of economic reasons. In China, 12% of the poorest had to forgo hospitalisation due to economic hardships, while less than 5% of the richest respondents had to do so [10]. Moreover, issues of CHE resulting from health services occurred as well, which have become especially prominent among vulnerable populations due to their poor economic situation. At the same time, unsatisfied financial protection capacity of medical insurances, especially fragmentation in health insurance, has come under the fierce public attack. Li and Wu’s study showed that the incidence of CHE among households covered by NCMS is 1.57 and 1.74 times higher than for households covered by UEBMI and URBMI, respectively [6]. Moreover, the incidence of CHE among rural households is almost 1.5 times higher than the national average [11]. On the other hand, the benefit of health services could increase the likelihood of residents receiving inpatient service utilisation, especially for the comparatively rich groups, which in turn may increase the risk of incurring CHE for the vulnerable groups [12].

Fragmentation in health insurance is mainly reflected in two features. First, the three health insurance schemes are administered and operated independently by different departments: the Chinese Ministry of Health is in charge of administering NCMS, and the Chinese Ministry of Human Resources and Social Security for URBMI and UEBMI. Second, these three insurance schemes were designed for different groups with different capacities to pay, which resulted in various financing mechanisms and payment standards. NCMS funds are pooled at the county level (2489 rural counties in 2013) [13]; the NCMS per capita fundraising amount was approximately ¥370.6 [14], and personal payment accounted for 18.09% [15]. While, the URBMI and UEBMI funds are pooled at the municipal (prefecture) level (333 municipalities/prefectures in 2013) [13]. The premium for UEBMI was 8% of the salary (6% paid by employers and 2% paid by employee). The average fundraising for URBMI was ¥360, and individual payments accounted for 21.67% [15]. These features have caused significant inequity across the 2489 NCMS, 333 UEBMI, and 333 URBMI schemes [16]. The compensation levels of URBMI and NRCMS are far below that of UEBMI. Compared with urban residents, the disparity of funds has had a greater impact on individuals in rural communities accessing and using health care. In addition, the reimbursement levels and benefit packages are inconsistent between districts. In fact, the reimbursement under NCMS was approximately 10% lower than for the other schemes [17].

For millions of migrants, this fragmentation severely affects their access to health services. Conservative estimates for health insurance participants in 2010 showed that over 100 million residents were covered by more than one scheme, leading to wasteful duplication of services and a loss in fiscal subsidies amounting to over RMB 12 billion [18]. As a result, actual coverage rates may be distorted due to duplicate insurance [19].

To address this issue, a nationwide policy pilot reform aiming to integrate fragmented medical insurance schemes was initiated. In 2016, the China State Council mandated the integration of basic medical insurance systems among urban and rural residents to merge the previously scattered small funds into a much larger risk pooling fund so as to make it more powerful to resist the bankruptcy risk of insurance funds, as well as to provide more financial protection to the insurant and reduce the inequity caused by household type [20]. Due to the inherent difficulties in integration, URBMI and NCMS, which have roughly similar premiums and institutional frameworks, were chosen to be merged as a preliminary step, which was named Urban and Rural Resident Basic Medical Insurance (URRBMI). A few districts in China pioneered the integration of the rural and urban schemes before the national guidelines were issued, and an even smaller number of districts (Dongguan and Zhongshan) directly merged the UEBMI, URBMI, and NCMS. Most regions chose the preliminary integration, merging only URBMI and NCMS, and unifying administration, premiums, reimbursements rates, and benefit packages. The principle upheld was that the benefit of new integrated schemes would follow higher reimbursement rates and wider benefit coverage among the former schemes. Most pilots set up more than one financing level as an interim measure for the participants to choose freely to achieve a final uniform. Further, the range of choice for rural residents among different designated medical institutions was expanded; residents could seek medical services in higher-rank hospitals like urban residents.

At present, China is implementing URRBMI nationwide. Integration reform is currently at a critical period, therefore, summaries of experiences with the process and evidence of its benefits are needed. Previous studies have begun to explore the effect of practices in integrated pilot programs, Chinese scholars described the practice, experience and summarizes of in integrated pilots through the qualitative method [21, 22]. In quantitative research, some studies investigated satisfaction of integration reform regionally or nationally [19, 23, 24]. As the most important purpose of integration reform, very few studies on equity were conducted to evaluated the realization of reform target, currently these studies mainly focused on health service utilization [10, 25, 26] or net benefit [27] However, as the final goal of health systems, hardly any studies focused on financial protection, and use national representative data to evaluate the effect of medical insurance integration reform in reducing CHE equity. Policy makers and the general public desperately wish to know that whether the following questions could be addressed or not: (1) Can health insurance integration decrease the overall incidence of CHE and improve equity among the target population as suggested by the theory? (2) What potential problems hinder the integration process? This study estimates CHE inequity for URRBMI, using URBMI/NCMS as reference points. Our findings will provide evidence for stipulating more targeted intervention policies for reducing age-long and aggravated inequitable issues in health insurance system that narrows the disparities of economic protection capacity among different groups under the same health insurance.

## Method

### Data collection and sampling method

**Fig. 1:**
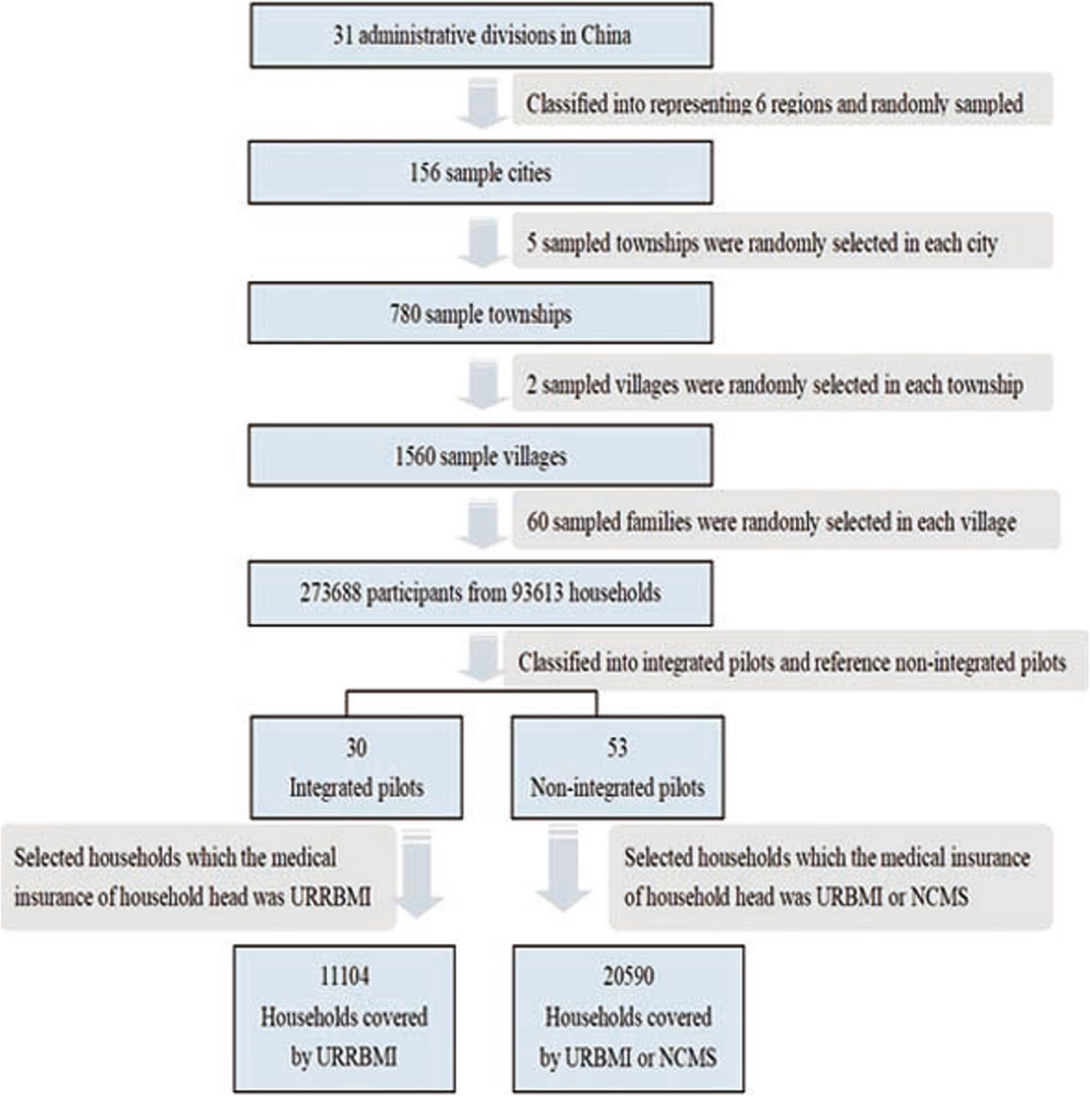
Data collection and sampling process

The dataset we use is obtained from the 5th China National Health Service Survey (NHSS) conducted in 2013. A multi-stage, stratified-cluster, random-sampling method was employed to generate a nationally representative sample of the whole population. Ultimately, 273,688 participants from 93,613 households were selected; the effective response rate was 82.1%. In order to estimate household CHE and income-related inequity covered by the different insurance schemes, we chose the pilot programs according to the implementation of the local health insurance-integration policies before the 5thNHSS. Households in areas that had implemented the pilot integration reform were considered as policy integration areas. If sample areas of provinces/municipalities/autonomous regions all implemented the integration policy, they were defined as integrated pilots; all sample areas of other non-integrated provinces/municipalities/autonomous regions that had similar per capita GDPs in 2012 were selected as reference non-integrated pilots. If partial sample areas of provinces/municipalities/autonomous regions implemented the integration policy, the rest sample areas were selected as reference non-integrated pilots. As a result, surveys were from 30 integrated pilots in 156 sample cities, belonging to 13 administrative divisions at a national level. Among these administrative divisions, there were three administrative divisions (Tianjin, Chongqing, Ningxia) where each sample city was identified as integrated pilot. We then selected administrative divisions (Beijing, Shanxi, Hebei) by per capita GDP in 2012, and all sample cities of these provinces/municipalities were used as reference non-integrated pilots. In the other 10 administrative divisions with integrated pilots, where part of the sample areas had implemented integrated pilots, the rest of the sample areas were reference non-integrated pilots. We selected households for which the head of household’s health insurance was URRBMI from 30 integrated pilots and chose households for which the head of household’s insurance was either URBMI or NCMS from 53 non-integrated pilots. Ultimately, 11,104 (35.0%) and 20,590 (65.0%) households covered by URRBMI and URBMI/NCMS, respectively, are considered here (Fig. 1).

### Variables

The NHSS dataset provides information on a wide range of general status, health status, and health service utilization items by households using a standardized questionnaire. In this paper, the dataset provides household information, including household size (≤2/3–4/≥ 5), residential area (urban/rural), region (east/middle/west), preferred health facilities (primary hospital/non-primary hospital), economic group quintile (annual household consumption expenditure was ranked into quintiles after adjustment for standard household size), presence of at least one household member aged 60 and over or 5 and under (yes/no), at least one household member with a chronic disease (yes/no), at least one hospitalized household member (yes/no), and the nature of household consumption expenditures in the past year, such as total household consumption expenditures, OOP health expenditures, and food expenditures. Apart from these variables, the dataset provides information on the household’s socio-economic background, including member gender (male/female), marital status (married/other), educational level (none/primary school/junior high school/high school or above), employment status (employed/retired/other), and type of medical insurance scheme (URRBMI/URBMI/NCMS).

### Measurements

#### Catastrophic health expenditures

CHE were calculated based on the method recommended by WHO based on their definition of CHE [2]. Following Xu et al. [28], we consider OOP health expenditures (direct medical spending) “catastrophic” if they are equal to or greater than 40% of the household capacity to pay (CTP). CTP is defined as household consumption expenditures minus basic subsistence needs spending adjusted for household size. The subsistence spending of each household was calculated according to the poverty line multiplied by standard household size. The poverty line refers to average annual food expenditure of a household whose food expenditure share of total household consumption expenditure is between the 45th and 55th percentiles of the entire sample. If food expenditure was less than subsistence needs spending, we use non-food household expenditures to represent household CTP and 40% as a threshold standard for CHE, but we also used 20, 30, 50, and 60% as threshold levels for sensitivity analysis. The percentage of households experiencing catastrophic health expenditure is called catastrophic head count (H_cat_), which is calculated as follows:

$$ {H}_{cat}=\frac{1}{N}\sum \limits_{i=1}^N{E}_i $$

where *N* presents the sample size and *E*_*i*_ = 1 When OOP payment of the *i*^*th*^ household exceed the threshold; otherwise *E*_*i*_ = 0. In order to measure the intensity of CHE, the mean gap (G_cat_) and mean positive gap (MPG_cat_) reported by Wagstaff and Doorslaer [29] are employed. G_cat _measures the average extent to which OOP health expenditure crossed the given catastrophic threshold in all sample families, which reflects the serious societal repercussions of CHE. MPG_cat _indicates the CHE burden of exceeding the given threshold for households experiencing CHE. The formulae are respectively as follows:

$$ {G}_{cat}=\frac{1}{N}\sum \limits_{i=1}^N{O}_i $$

where *O*_*i*_ presents the proportion by which the *i*^*th*^ household exceed the threshold.

$$ MP{G}_{cat}=\frac{H_{cat}}{G_{cat}} $$

### Poverty and impoverishment

If the total expenditure of a household was less than subsistence spending, it was categorized as poor. Impoverishment is defined as a non-poor household fall into poverty after health service payment. When a household’s total expenditure was more than subsistence spending (adjusted for household size) and the total expenditure was less than subsistence spending after health service payment, the household was categorized as impoverished.

### Concentration index

The concentration index (CI) has been widely used in studies on CHE inequity conducted by the World Bank [30] and is computed by the standard method provided by the “convenient covariance” formula [11]. CI ranges between − 1 and + 1 [29]. In the absence of inequalities, the value of the concentration index should be zero. We used CI to analyze the income-related inequity of CHE incidence (C_E_) in the surveyed pilots. The index took a negative (positive) value if C_E_ disproportionately concentrated among the poor (high-income) groups, respectively. The larger the absolute value of the concentration index indicated, the greater the inequality in CHE incidence. It is calculated as:

$$ CI=\frac{2}{\mu}\operatorname{cov}\left({y}_i,{\gamma}_i\right) $$

where *y*_*i*_ represents CHE rate of household and *γ*_*i*_ represent the household’s fractional rank by economic status.

### Concentration index decomposition

After CI is calculated, a decomposition method based on probit model is applied to assess the major contributing factors to the observed inequality [29]. The decomposition approach enables us to quantify each factor’s contribution to income-related inequity associated with CHE.

$$ y={a}^m+{\sum}_j{\beta}_j^m{x}_j+\varepsilon $$

where *y* represents whether households experience CHE. In the regression, ${\beta}_j^m$ is the partial effect of each variable and evaluated at sample means; *α*^*m*^ is the constant term; *ε* is the error term. On the basis, CI decomposition is conducted.

$$ CI={\varSigma}_j\left({\beta}_j^m{\overline{x}}_j/\mu \right){C}_j+G{C}_{\varepsilon }/\mu $$

Where *μ* is the mean of dependent variable, $\left({\beta}_j^m\overline{x_j}/\mu \right)$ is the elasticity of each *x*_*j*_, *C*_*j*_ is the concentration index of *x*_*j*_, $\overline{x_j}$ is the mean of *x*_*j*_, the first term of the Equation is the contribution of observable variables, the last term is the generalized concentration index of ε.

### Statistical analysis

SAS 9.4 software was used for general descriptive analysis and CHE analysis. Stata 11.0 software was used to measure and decompose CHE inequality.

## Results

### Description of survey population

Table 1 shows the demographic information of the 11,104 and 20,590 households covered by URRBMI and URBMI/NCMS, respectively. Many of the statistics between them are similar, only two main differences between the demographics. URRBMI and URBMI/NCMS populations differ in which region has the most households (eastern [53.09%] and middle [37.08%]), and degree of income levels.

**Table 1:** Characteristics of participants covered by URRBMI, URBMI or NCMS in 2013

	Households covered by URRBMI		Households covered by URBMI or NCMS	
	*N*	%/mean	*N*	%/mean
Household size				
< =2	4860	43.77	9210	44.73
3–4	4342	39.10	8347	40.54
> =5	1902	17.13	3033	14.73
Gender of household head				
Male	8659	77.98	15,691	76.21
Female	2445	22.0	4899	23.79
Marital status of household head				
Unmarried and others	9273	83.51	17,433	84.67
Married	1831	16.49	3157	15.33
Educational level of household head				
Illiterate	1773	15.97	2838	13.78
Primary school	4206	37.88	7435	36.11
Junior middle school	3955	35.62	7859	38.17
Senior high school or above	1170	10.53	2458	11.94
Employment status of household head				
Employed	8333	75.0	16,427	79.78
Retired	440	4.0	755	3.67
Unemployed and others	2331	21.0	3408	16.55
Place of residence				
Urban	3439	30.97	6234	30.28
Rural	7665	69.03	14,356	69.72
Region of household				
East	5895	53.09	5737	27.86
Middle	1583	14.26	7635	37.08
West	3626	32.65	7218	35.06
Type of medical insurance scheme				
URRBMI	11,104	100	–	
MIUR	–		2715	13.19
NCMS	–		17,875	86.81
Preferred institution for common diseases				
Primary hospital	9718	87.52	19,051	92.53
Non-primary hospital	1386	12.48	1539	7.47
Household including hospitalized member				
Yes	2312	20.82	4592	22.30
No	8792	79.18	15,998	77.70
Household including member with chronic disease				
Yes	5042	45.41	8550	41.53
No	6062	54.59	12,040	58.47
Household with members aged five or younger				
Yes	2043	18.40	4169	20.25
No	9061	81.60	16,421	79.75
Household with members aged sixty and above				
Yes	5188	46.72	8897	43.21
No	5916	53.28	11,693	56.79
Expenditure quintile ^a^ (US$)^b^				
Quintile I (poorest)		417.58		384.02
Quintile II		855.55		778.51
Quintile III		1365.04		1201.88
Quintile IV		2114.28		1831.15
Quintile V (wealthiest)		4801.56		3816.38

*US$* United States Dollar

^a^Quintile 1 is the poorest 20%, and quintile 5 is the wealthiest 20%

^b^According to the exchange rate of 6.1932 yuan to US$ 1.00

### The incidence and intensity of CHE at different threshold level under URRBMI and URBMI/NCMS

**Fig. 2:**
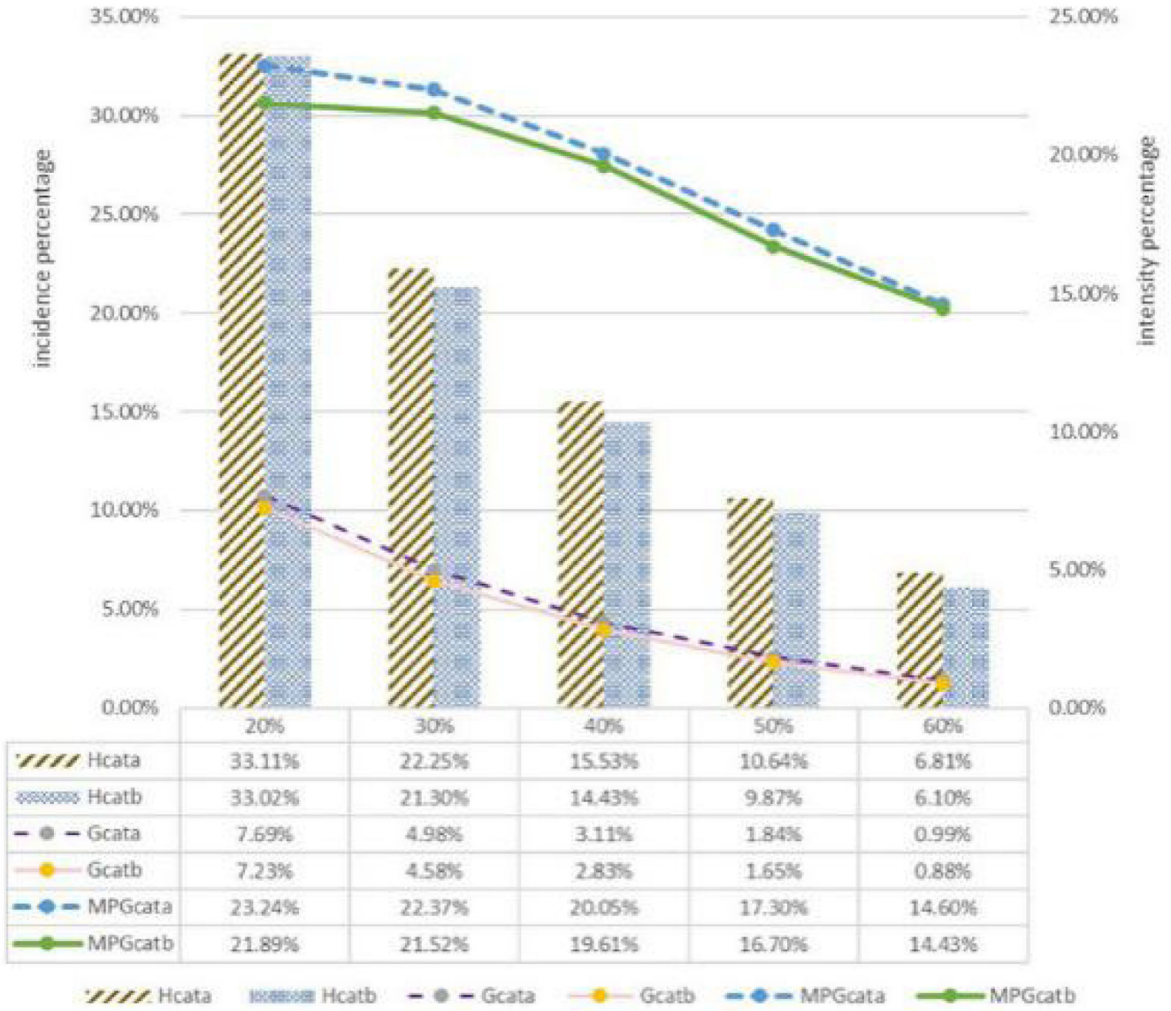
H_cat_, G_cat,_ and MPG_cat_ at different threshold level under URRBMI and URBMI/NCMS. **a**. integrated URRBMI scheme, **b**. non-integrated schemes (URBMI/NCMS). Note: Quintile 1 is the poorest 20%, and quintile 5 the wealthiest 20%

As Fig. 2 showed, the overall H_cat_, G_cat_, and MPG_cat_ for both URRBMI and URBMI/NCMS fell as thresholds increased. Statistics from the integrated scheme showed higher CHE incidence except at the 20% threshold (*P* < 0.05). At the 40% standard, the incidence of CHE under URRBMI (15.53%) was 1.10% higher than for families covered by URBMI or NCMS (14.43%). At the same threshold, G_cat_ and MPG_cat_ under URRBMI households were higher than for URBMI or NCMS.

### Distribution of catastrophic incidence and intensity across consumption expenditure quintiles under URRBMI and URBMI/NCMS

**Fig. 3:**
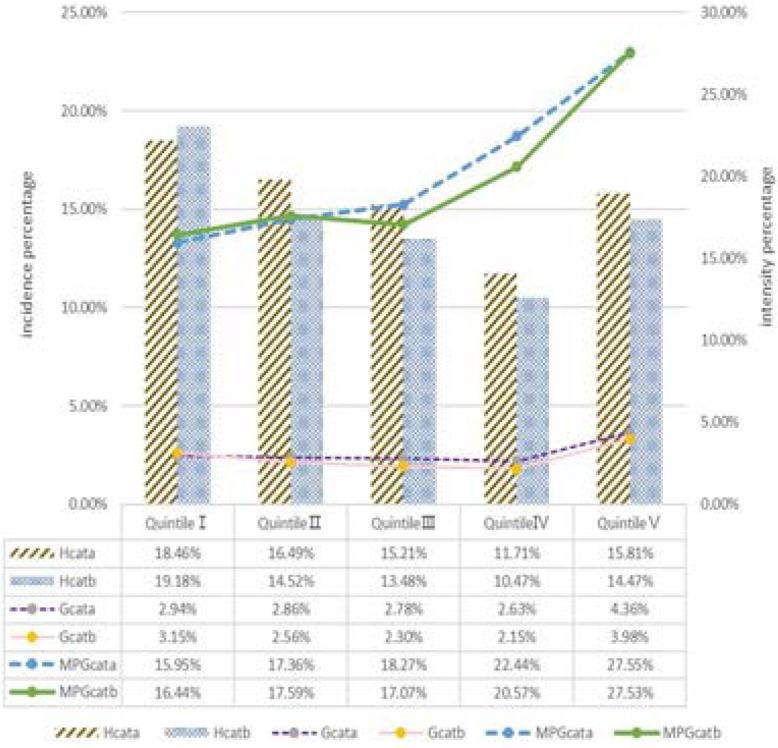
Distribution of H_cat_, G_cat_ and MPG_cat_ across consumption expenditure quintiles (40% threshold). **a**. integrated URRBMI scheme, **b**. non-integrated schemes (URBMI/NCMS). Note: Quintile 1 is the poorest 20% and quintile 5 the wealthiest 20%

Further, Fig. 3 provides additional insights into different expenditure quintiles under the 40% threshold. Both schemes showed that CHE incidence in the poorest group was the highest, followed by the second-poorest group; the value of second-richest group was the lowest. H_cat_ of URRBMI households in the second-poorest group was higher than for URBMI or NCMS families (*P* < 0.05), while there was no significant statistical difference between integrated and non-integrated scheme in other quintile groups. MPG_**cat**_ values increased with the improvement in family economic level in both schemes. Compared with households covered by URBMI or NCMS, households covered by integrated URRBMI had lower G_cat_ among the poorest group and lower MPG_cat_ among the poorest and the second-poorest group. But in other groups, the situation was opposite. The G_cat_ and MPG_cat_ gap between integrated and non-integrated scheme was especially obvious in quintile IV and quintile III. The highest G_cat_ and MPG_cat_ occurred in the wealthiest group in both schemes but were higher in URRBMI.

### Out-of-pocket payment for health service and impoverishment

The higher the economic level of household, the more OOP and CTP they have. Whether from an overall view or in each quintile group, the average OOP and CTP of URRBMI households were higher than URBMI or NCMS (*P* < 0.05), and the gap of OOP or CTP were respectively up to $347.44, $1903.09 in the Quintile V group. Participants covered by URRBMI had 0.34 and 0.29% lower fraction of OOP occupied CTP compared to URBMI or NCMS in the poorest group and the second-richest group, respectively. Without considering OOP payment, the total proportions of poor households were 3.36% higher under integrated scheme than unintegrated (*P* < 0.05). Poor households covered by these two schemes only concentrated in the poorest and second-poorest groups, and the rate in the poorest was particularly severe (exceeding 50%). However, after health service payment, each economic group was impoverished, and notably, the impoverished rate for URRBMI households in the poorest group was 2.72% lower than that for URBMI or NCMS, but the situation was reversed for Quintile III and Quintile IV groups (Table 2).

**Table 2:** Distribution of household health expenditure characteristics and impoverishment level across consumption expenditure quintiles

	Households covered by integrated URRBMI						Households covered by URBMI or NCMS					
	Quintile I	Quintile II	Quintile III	Quintile IV	Quintile V	Total	Quintile I	Quintile II	Quintile III	Quintile IV	Quintile V	Total
Average OOP (US$)^a^	151.03^*^	306.03^*^	445.92^*^	578.84^*^	1609.75^**^	618.08^**^	137.45	265.79	391.67	520.54	1262.31	515.27
Average CTP (US$)^a^	703.58^**^	1575.86^**^	2538.98^**^	3946.78^**^	8519.03^**^	3455.17^**^	648.00	1414.97	2230.41	3396.60	6615.94	2859.38
OOP/CTP (%)	22.84	20.65	18.55	15.76	17.64	17.89	23.18	19.91	18.48	16.05	17.30	18.02
HH with poverty (%)	64.71^**^	9.50^**^	0	0	0	14.88	55.10	2.48	0	0	0	11.52
HH with IM (%)	7.50^**^	13.65	6.09^**^	2.88^*^	1.94	6.41	10.22	12.94	3.90	1.70	1.48	6.07

*OOP* out of pocket, *CTP* capacity to pay, *HH* household, *IM* impoverishment, *US$* United States Dollars

^a^According to exchange rate of 6.1932 yuan to US$ 1.00

^b^Quintile 1 is the poorest 20%, and quintile 5 is the wealthiest 20%

* indicates that the difference was statistically significant **P < 0.01 ***P < 0.001

### Inequality of catastrophic health expenditure and its decomposition

URRBMI households had a lower concentration index (− 0.053) and fell 28.38% than URBMI/NCMS households (− 0.074), indicating that there was less inequality among the participants covered by URRBMI although its CHE still concentrated mostly in poor households.

Through the decomposition analysis, the three major indices in Table 3 were obtained, including marginal effect (*β*_*k*_), CI_k,_ and each determinant’s contribution to CI. *β*_*k*_ explains the association between each factor and CHE incidence, where a positive value means positive role in increasing probability of risk for CHE incidence, and a negative sign means a negative correlation; the larger the absolute value of *β*_*k*_, the higher the degree of association. CI_k_ denotes the distribution over wealth factor of each determinant; a positive CI indicates that the studied determinant is more prevalent among people of higher economic groups, while a negative value indicates that the studied determinant is more concentrated among poor groups. Contributions to CI refer to the relative contribution of each determinant; the sign represents positive or negative contributions to CHE inequality. Positive contribution means that the corresponding variables aggravate the inequality, and vice versa.

**Table 3:** Decomposition of inequality in CHE (40% threshold)

Variables	Households covered by URRBMI				Households covered by URBMI or NCMS			
	β_k_	*P*-value	CI_k_	Contribution to CI(%)	β_k_	*P*-value	CI_k_	Contribution to CI (%)
Household including hospitalized member								
Yes vs. No	0.248**	< 0.001	0.134	−84.53	0.216**	< 0.001	0.108	−48.21
Household including member with chronic disease								
Yes vs. No	0.076**	< 0.001	0.009	−3.63	0.080**	< 0.001	0.001	−0.17
Households including members aged above sixty years								
Yes vs. No	0.041**	< 0.001	− 0.120	28.27	0.045**	< 0.001	−0.131	23.54
Households including member aged below five years								
Yes vs. No	0.021*	0.045	0.025	−1.16	−0.004	0.52	0.028	0.24
Preferred institution grade for common diseases								
Primary hospital vs. Non-primary hospital	−0.014	0.14	−0.036	−5.53	− 0.018	0.047	− 0.024	−3.73
Family economic level quintile				28.65				37.28
Quintile II vs. Quintile I	0.009	0.30	−0.399		−0.017*	0.008	−0.398	
Quintile III vs. Quintile I	0.005	0.62	0.001		−0.016*	0.017	0.002	
Quintile IV vs. Quintile I	−0.021*	0.03	0.400		−0.044**	< 0.001	0.400	
Quintile V vs. Quintile I	0.001	0.93	0.800		−0.012	0.08	0.800	
Household size				45.41				18.58
< =2 vs. > = 5	0.173**	< 0.001	−0.070		0.127**	< 0.001	− 0.054	
3–4 vs. > = 5	0.082**	< 0.001	0.050		0.057**	< 0.001	0.055	
Gender of household head								
Male vs. Female	0.017*	0.02	0.002	−0.38	0.029**	< 0.001	−0.013	2.66
Marital status of household head								
Married vs. Unmarried and others	−0.017	0.043	0.038	6.68	−0.020*	0.002	0.037	5.66
Educational level of household head				41.71				22.08
Primary school vs. None	−0.029**	< 0.001	−0.057		−0.040**	< 0.001	− 0.080	
Junior middle school vs. None	−0.067**	< 0.001	0.101		−0.057**	< 0.001	0.089	
Senior high school or above vs. None	−0.074**	< 0.001	0.211		−0.055**	< 0.001	0.243	
Type of medical insurance scheme								
NCMS vs. URBMI	–	–	–	–	0.033**	< 0.001	− 0.030	8.04
Region of household				3.59				3.77
East vs. West	−0.040**	< 0.001	0.020		−0.010	0.07	0.050	
Middle vs. West	−0.067**	< 0.001	− 0.013		− 0.029**	< 0.001	0.025	
Employment status of household head				18.93				12.07
Employed vs. Unemployed and others	−0.068**	< 0.001	0.025		−0.079**	< 0.001	0.019	
Retired vs. Unemployed and others	−0.040*	0.002	0.173		−0.015	0.16	0.212	
Residence								
Rural vs. Urban	0.037**	< 0.001	−0.077	24.41	0.023**	< 0.001	−0.056	8.51

* indicates that the difference was statistically significant *P < 0.05 **P < 0.01

Among households covered by URBMI/NCMS and URRBMI, the total contribution percentage was 90.32%. and 102.42%, respectively, which indicated that the 9.68% negative contribution in URBMI/NCMS and 2.42% positive contribution in URRBMI to CHE incidence inequity is explained by the error term of the regression. The contribution to inequity in URBMI or NCMS households was mainly associated with having hospitalized member (− 48.21%), which increased the possibility of CHE among the rich (CI = 0.108), reducing inequality. The remaining factors were economic status (37.28%), having household member aged 60 or above (23.54%), education status of household head (22.08%), and household size (18.58%).

The contribution to inequity in URRBMI households can be explained by the following determinants: residence (24.41%),which emerged its contribution in increasing inequity under integrated scheme, and following factors consistent with URBMI or NCMS: having hospitalized member (− 84.53%), household size (45.41%), education level (41.47%), economic status (28.65%), having a household member aged 60 or above (28.27%).

For both schemes, having hospitalized member, member aged 60 or above, smaller household size, and lower education level, as well as better economic and employment status, all had higher prevalence in CHE. Households with hospitalized member were most concentrated among the rich. Those with member aged 60 or above (CI_k_ = − 0.120) and smaller household size (CI_k_ = − 0. 070) tended to happen to the poor (Table 3).

## Discussion

Measuring CHE occurrence and its equity is important for identifying the existing problem of integration reform and effective ways for further improving it. It also measures the distance to achieve universal health insurance. This study compared incidence and intensity of CHE and equity of incidence under integrated and non-integrated schemes, and the results are discussed.

### Integrated health insurance performed poorly in total CHE incidence, intensity and impoverishment, but it performed more effectively among the disadvantaged population

The total CHE incidence, intensity and impoverishment for households covered by URRBMI was more severe than that with non-integrated, improvement of health service availability in low-income and middle-income countries could also result in more households experiencing CHE [28]. which might partly be explained by URRBMI in promotion of utilizing more health services due to its extended benefit package, besides, URRBMI participants prefer to seek more service in high-level hospitals, which tends to incurring higher medical payment [31]. In both integrated and non-integrated schemes, the highest G_cat_ and MPG_cat_ occurred in the wealthiest groups, offering policymakers a stark reminder that although those relative wealthier groups had lower CHE incidence, but once CHE happens, it is more devastating, and in this regard, it was more severe in URRBMI. In addition, URRBMI performed more effectively with lower G_cat_ and impoverishment among the poorest group and lower MPG_cat_ among the poorest and second-poorest groups. One possible explanation was that most URRBMI households were concentrated in the eastern region with relatively developed economy, as result the indicators were lower owing to better overall economic level of the poor or better governance of poverty. However, the lower fraction of OOP occupied CTP in the poorest group under URRBMI indicated that the integration reform greatly helped to relieve catastrophic payment burden and impoverishment. A study considering inter-annual variation also demonstrated that the security level among the low income group improved significantly compared with the high income group owing to the integrated reform [32].

### A slightly better performance in equity of CHE displayed in integrated schemes, but the disadvantaged populations remain vulnerable

According to our CI calculations for URRBMI households, the absolute value under URRBMI was 28.38% lower than URBMI/NCMS. Thus, integrated insurance led to a modest equity improvement. The reason for insurance integration facilitating equity may partly be explained by its unified management, higher-level benefit package, and reimbursement rate which plays a positive role in reducing the existing structure disparities. On this point, its progress is commendable. However, it must be noted that people of lower economic status were still more vulnerable to CHE, as the CHE rate of the poorest remains 1.17 times higher than the wealthiest.

### Financing and health resource disparity hinder equity

The factor of residence plays a positive role in increasing inequity in the integrated URRBMI, its contribution occupied a lot (24.41%) in all above determinants. Under URRBMI, the gap of CHE rate between rural and urban areas did not improve. As a scheme aimed to narrow the disparities between urban and rural areas as well as protecting urban and rural residents from poverty due to illness and promoting the fair use of health service, this result indicated that the equalizing goal of the URRBMI has not been reached. In addition, the inequality inherited from the two independent schemes is magnified as compared with non-integrated scheme, as prior studies indicated [10].

A real “fusion” has not taken place because there is a gap in financing level between urban and rural areas. Among the integrated provincial and municipal areas nationwide, most regions set several financing levels in one scheme [33–35]. The standard of reimbursement and benefit package depends on the standard and level of insurance premium; thus the higher the level of financing, the better the service obtained [36]. Although the measure takes into account the differences in payment capacity between rural and urban areas, CHE inequality remains unsolved, as rural residents tend to choose lower financing level, whether limited by income or influenced by past habits [37, 38]. Under the differentiated financing model, together with a lack of sufficient measures to address economic burdens faced by different social groups, and to address difficulties in defining appropriate premiums levels, rural residents failed to get better reimbursement owing to underutilized health services. However, urban residents tended to receive more health services and enjoy better welfare. This leads to reverse subsidy [39, 40] and thus resulted in a new inequality. As more and more provinces started to unify financing levels, a more sustainable approach lies in enhancing the insurance scheme’s reimbursement for the disadvantaged, while replacing a voluntary participation with mandatory requirement, besides, appropriate level of financing must be developed and gradually increased.

After integration, former fixed-point medical institutions under URBMI/NCMS were unified into the management scope of the URRBMI, and this has led to more choices for medical institutions. Improvement in URRBMI’s convenient off-site medical billing may increase rural patients going to urban hospitals. Increasing hospitalization rates in tertiary medical institutions and decreasing hospitalization rate in primary and secondary institutions after integration witnessed in western China, which support the findings of this study [41]. Pooled rural and urban resources are unevenly distributed and high-quality resources are mostly concentrated in urban areas [42, 43]. Wide disparities in quality and quantity of health technicians, material resources allocation, and government financing continually increased [14]. Rural residents are prompted to use health services at this opportunity and accordingly enormously increase their medical costs. Therefore, improving the hierarchical medical system is imperative. More support must be provided to improve the quality and capacity of rural areas that lack medical resources.

### The phenomenon of the wealthy households using more hospitalization service has not been improved

In URRBMI, inpatient service utilization concentrated more among the rich, which is consistent with the findings of many researches [44–46]. Having hospitalised household member in both schemes was the greatest factor in reducing CHE inequality by producing more inpatient service utilisation among the rich; that is to say, the nature of CHE inequity reduction pertains to the inequity of hospitalisation services that the rich use more than the poor. In both schemes, the proportion of households using inpatient services gradually increased with economic level. Compared with households covered by URBMI/NCMS, the floating scope among URRBMI households with different economic groups was larger as a whole. This is especially obvious between the richest and the poorest; the disparity of hospitalisation rate between Quintile1 and Quintile 5 in URBMI/NCMS households was 11.92%, while it was up to 15.12% in URRBMI. Meanwhile in the URRBMI scheme, underutilisation of inpatient services among the poor households is more highlighted; in our study, the value of the poorest households covered by URRBMI was 1.24 times lower than that of URBMI/NCMS households significantly (see Additional file 2). The hospitalisation utilisation of the poorest did not seem to improve under the integrated system and it was due to several reasons. First, due to unawareness of the benefits of the integrated scheme, hospitalization service was not well-promoted for the disadvantaged; a survey in China also confirmed that most rural residents were not clear that they were participating in the merged health insurance rather than NCMS [47, 48]. Second, NCMS and URBMI were designed for inpatient and outpatient catastrophic disease, after integration, the situation that limited diseases and reimbursement ratios covered by the insurance policies at outpatient clinics were far below inpatient service in most areas has not been solved well, so that for some diseases suitable for outpatient treatment, medical expenditures were only reimbursed after using hospitalization services. People with higher income tended to be more inclined to utilize hospitalization services and easy to occupy the resource of the poor. The benefit packages for outpatients should be improved, and what’s more, pay attention to avoid the economic risks caused by the release of needs in inpatient and outpatient care.

### The sign of demographic factors contribution to CHE inequality must be noted and addressed in the reform process

Demographic factors like employment, education level, economic status, household size, and having members aged 60 or above still played positive roles in increasing CHE inequity among the poor covered by URRBMI. It is clear that smaller households and lower economic status are associated with a higher proportion of CHE although they were covered by the same benefit package [49] because risks were shared by fewer people and their lower medical cost affordability [50]. Thus, improving the small-scale household’s anti-risk ability, narrowing the gap in economic status by consolidating taxation policies to redistribute income, as well introducing policies to support the disadvantaged [51] could be helpful in this regard. In addition, households with members aged 60 or above with a higher risk of experiencing CHE were most concentrated among the poor, consistent with earlier studies [49, 52, 53]. It is worth noting that the likelihood of new and costly non-communicable diseases increases with living a longer life [54]. Therefore, it is necessary to increase financial protection capacity to reduce CHE proportion in poor and elderly households. For households with disadvantaged human capital, only by the effort of reforming insurance scheme is not enough, other supportive policies such as assisting the poor with more income improvement initiatives should be encouraged, it is not until a more comprehensive strategies that targeted at the multi-vulnerability that can we provide the long lasting protection for those most disadvantaged people.

According to our findings, the following recommendations should be considered. First, there should be a promotion of a more precise insurance intervention policy that targets the most vulnerable population; this includes the poor, aged, as well as those living in rural areas, and families that are impoverished due to catastrophic medical expenditure. These populations must be provided with free insurance premium, higher reimbursement rate, less self-co-payment, or fee exemption after exceeding the ceiling. The burden to allocate additional resources to address the inequity in the catastrophic rate is manageable. The additional cost gap for promoting this policy can be addressed through the following measures: 1) merging small insurance funds into a larger fund, such as upgrading the current municipal level fund into a provincial or even larger regional level fund; 2) unifying financing levels within all URRBMI in different regions; 3) requiring mandatory participation for all URRBMI enrolees; 4) providing subsidies or exemptions for drug or treatment expenses for certain catastrophic diseases, or lifting the ceiling or decreasing deductibles for those who are eligible for the catastrophic disease compensation scheme. The introduction and implementation of this policy needs diversified financing channels, and a dynamic adjustment mechanism of financing and benefits to ensure the sustainability of the health insurance fund. This will prompt health insurance institutions to develop an accurate and proper actuarial system. Second, there is a need to improve the financial protection in outpatient by including costly diseases suitable for outpatient treatment into benefit packages, while increasing the reimbursement ratio and cancelling deductible lines. Whether in outpatient or inpatient services, it is important to control medical expenses by changing the payment mechanism. While the ways on how to unify existing payment methods nationwide is underway, the existing obstacles that hinder its smooth implementation should be explored and quickly dealt with. Third, the government should increase financial protection for the disadvantaged by devising comprehensive strategies, in addition to current unified non-discriminatory insurance arrangement. Stronger government financial support for the existing Medical Assistance System for the Poorest should be stepped up, and more favourable benefit packages targeted at the needs of vulnerable people should be designed and implemented efficiently. It is also of great importance to create jobs or provide different types of subsidies for the disadvantaged. In the end, indicators for evaluating the effectiveness of targeted policy interventions should be established and monitored.

There are several limitations in this study. First, it is a cross-sectional study, thus the causal relationship between the integration reform and measured factors could not be proved, there possibly existing some biases in the evaluation of the effects of integration reform. Second, the data was from the fifth health service survey, which was collected through respondents’ self-reported, which could be affected by memory biases; Third, until this research was conducted, some integrated areas were in the initial stage of insurance integration reform, maybe the policy intervention effect was not obvious despite the fact that this study present the average situation of nationwide; Fourth, in this study, the periods for policy implementation were not singled out as a useful variable into the analytical model, which make us unable to interpret the time effect of the policy pilot. Thus, it is more advisable to add the length of policy implementation as a factor in future studies. Fifth, some respondents can’t afford health service payment maybe missing, which might lead to the under estimation of CHE to some extent.

## Conclusions

The effect and problems which integrated URRBMI reform coexist. Overall, households covered by URRBMI are at a higher risk of incurring CHE, but the intensity and impoverishment has improved for the poorest, the slightly better performance in equity of CHE was displayed but the possibility of CHE risk still concentrated among the poor. Measures should be considered by promoting more precise insurance intervention policies targeted at most vulnerable population like providing free insurance premiums, higher reimbursement rates, less self-co-payment or fee exemption after exceeding the ceiling, and improving the financial protection of outpatients by including costly diseases suitable for outpatient treatment into benefit packages. It is not enough to improve integration reform itself; comprehensive strategies to increase financial protection to the disadvantaged are also required, such as providing favourable targeted benefit packages or creating jobs. Strict monitoring and supervision are necessary during implementation. Future studies should discuss that on the basis of breaking the limits of household registration whether thoroughly integrating UEBMI, URBMI, and NCMS is more conducive to CHE fairly distribution over wealth factor.

## Supplementary information

**Additional file 1.** Description of the URRBMI and URBMI/NCMS household involved in the study.

**Additional file 2:Figure S1.** Distribution of proportion of inpatient service utilization among households across consumption expenditure quintiles under URRBMI and URBMI/NCMS.

## Data Availability

Datasets used during the current study are available from Centre of Health Statistics and Information, National Health Commission of the People’s Republic of China. Owing to the confidential policy, the data are not publicly available. Requests for the dataset should be directed to Centre of Health Statistics and Information, National Health Commission of the People’s Republic of China.

## Abbreviations

CHE: Catastrophic health expenditure
CI: Concentration index
CTP: Capacity to pay
G_cat_: Mean gap
IM: Impoverishment
MPG_cat_: Mean positive gap
NCMS: The New Rural Cooperative Medical Scheme
NHSS: National Health Service Survey
OOP: Out of pocket
UEBMI: Urban Employee Basic Medical Insurance
URBMI: Urban Resident Basic Medical Insurance
URRBMI: Urban and Rural Residents Basic Medical Insurance
WHO: World Health Organization
