# Supplementary material for: Can the reform of integrating health insurance reduce inequity in catastrophic health expenditure? Evidence from China
Source: Int J Equity Health. 2020 Apr 3;19:49. doi: 10.1186/s12939-020-1145-5 (PMC7126184; doi:10.1186/s12939-020-1145-5)
Supplement: Supplementary file 2 — Additional file 2:Figure S1. Distribution of proportion of inpatient service utilization among households across consumption expenditure quintiles under URRBMI and URBMI/NCMS. [file 12939_2020_1145_MOESM2_ESM.docx]

**Additional file 2: Distribution of proportion of inpatient service utilization among households across consumption expenditure quintiles under URRBMI and URBMI/NCMS.**


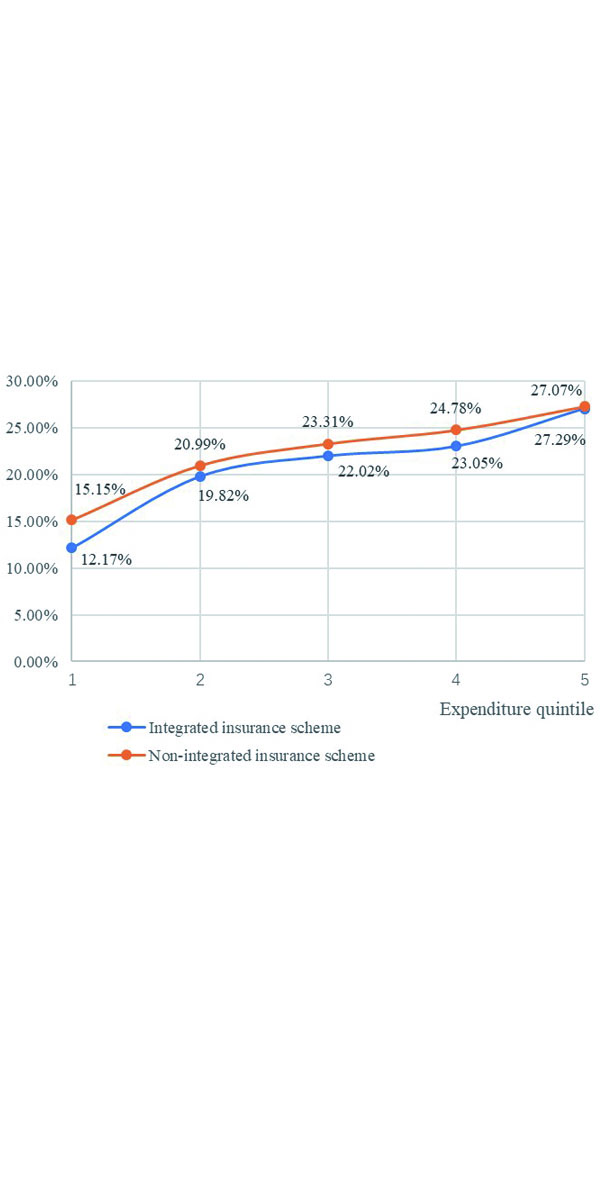


Figure S1 Distribution of proportion of inpatient service utilization among households across consumption expenditure quintiles under URRBMI and URBMI/NCMS.
